# Laryngeal Trauma, Its Types, and Management

**DOI:** 10.7759/cureus.29877

**Published:** 2022-10-03

**Authors:** Abhinav Malvi, Shraddha Jain

**Affiliations:** 1 Department of Otorhinolaryngology and Head and Neck Surgery, Jawaharlal Nehru Medical College, Datta Meghe Institute of Medical Sciences, Wardha, IND

**Keywords:** penetrating laryngeal trauma, blunt laryngeal trauma, thyroid cartilage fracture, sharp weapon injury, laryngeal trauma, laryngeal injury

## Abstract

Laryngotracheal wounds are rare; however, they have a significant mortality rate. These wounds can be blunt or penetrating. Usually, the larynx is protected from blunt trauma by the sternum and jaw. A "clothesline" injury happens when the exposed neck is struck by a hard object, such as a wall wire or tree branch, or when an attack is intended to damage the larynx. Additionally, injuries may occur when the neck is stressed due to damage, such as in a rear-end accident that causes a whiplash-like injury or when the larynx is intentionally targeted for harm. Penetrating neck trauma may result in injury to the larynx.

Assume a patient has suffered a penetrating or severe neck injury. It is usually evident from their medical history or a quick trauma evaluation in that case. However, it is recommended to be cautious for anterior neck injuries in general and to have a low threshold for establishing a surgical airway. The priority is securing an airway when a patient with a laryngeal injury arrives in the emergency room. The operating surgeon may request any flexible laryngoscopy, computed tomography (CT), esophagram, and chest X-ray for additional examination, depending on the nature of the damage and the patient's health.

After the examination, the initial step in treating laryngeal injuries should be to locate and secure the airway. According to the evaluation and management based on the Schaefer classification system for laryngeal injury, the patient is treated based on whether the patient has impending airway obstruction or a stable airway. Medical management or observation and surgical management depend on the site and severity of the injury, patient condition, and type of injury. There are several complications related to laryngotracheal trauma, which can be minor or even fatal. Following successful treatment, postoperative and rehabilitative care, vocal rest, speech therapy, and swallowing therapy may be necessary.

## Introduction and background

Trauma to the larynx is rare but may prove to be fatal. There are two types of laryngeal trauma: penetrating and blunt. Laryngeal injuries may heal, even with a minor injury, fibrous union, a deformity, and impaired laryngeal function [1-9]. These wounds can be penetrating or blunt and can develop in the supraglottic, glottic, or infraglottic areas. A thorough grasp of laryngeal damage is necessary, and varying sequences and degrees of wound severity can occur with blunt rather than penetrating laryngeal trauma. Patients with a history of anterior neck trauma must have their larynx thoroughly examined for damage symptoms and have a secure airway confirmed before additional testing [10].

Tracheal trauma can be penetrating, blunt, acute, or subacute. A blow to the neck may cause an acute traumatic disruption of the trachea, a knife wound, or crush injuries to the upper chest, despite the possibility of subacute injuries. The trachea may be disrupted after a blunt neck injury, frequently within 3 cm of the carina [11]. Tracheal trauma may be followed by damage to adjacent structures, such as vascular damage to the main arteries, carotids, or jugulars, or involvement of the digestive system, according to etiology. However, early detection and surgical treatment are necessary to prevent complications and the loss of respiratory function [12].

## Review

Etiology

Motor vehicle accidents are the most common cause of laryngeal injuries. Even with seat belts, airbags, and different advancements in car security gear, the occurrence has been markedly reduced. Injuries similar to those from a clothesline, attack/assault, strangulation, execution, and penetrating wounds are some of the additional causes. Latrogenic laryngeal damage can occur during bronchoscopy, rising intubation, or percutaneous tracheostomy.

The sternum and jaw often protect the larynx from blunt injuries. When the exposed neck is struck by a hard surface, such as a wall wire or tree branch, or when an attack is intended to hurt the larynx, it results in a "clothesline" injury. Injuries can occur when the neck is damaged and put under stress. Injuries can also occur in a rear-end collision that results in a whiplash-like injury or when the larynx is intentionally targeted for harm. The larynx is susceptible to damage in penetrating neck injuries. It is usually evident from the patient's history or the vital trauma assessment if a patient has experienced a penetrating or traumatic neck injury. However, it is recommended to be vigilant for anterior neck injuries in general and to require only a small threshold to secure a surgical airway [10].

Epidemiology

Laryngeal injury forms less than 1% of all traumatic injuries. Despite being rare, they may be quite severe. After intracranial injuries, laryngeal injury is the second most common reason for death in patients with head and neck injuries. A laryngeal injury degree is rated in specific academic investigations using the Schaefer classification system (Table 1) [13]. 

**Table 1:** Schaefer Classification System for Laryngeal Injuries

Type of Injury	Classification System of Schaefer for Laryngeal Injuries
1	Minor hematoma of endolarynx or fracture less laceration
2	Extreme edema, hematoma, a fracture that is not dislocated or break in the mucosa in which cartilage is not exposed
3	Extensive edema, extensive break in the mucosa, dislocated fractures or immobility of vocal cord, exposed cartilage
4	Serious interruption of the anterior larynx, unsteady fractures, fracture lines, and severe trauma of mucosa
5	Total isolation of trachea and larynx [13]

It may provide a helpful basis for the medical professional. Most healthcare professionals classify patients' airways into stable or unstable groups in clinical practice since this has an immediate bearing on the consequent workup and assessment. Before further testing, unstable patients should have a stable airway secured, mostly by an emergency tracheostomy or cricothyrotomy. Stable patients may have additional testing using fiberoptic inspection.

Laryngeal trauma: Pathophysiology

Blunt Trauma of Larynx

The degree of severity is frequently proportional to the force used and the area across which it is applied. The larynx and trachea can develop structural malformations due to high-velocity traumas that fracture the cartilages that line the larynx and trachea. The "clothesline" injury, the most severe occurrence, occurs when a motorcyclist collides with a small, static item, like a fencing net or branch of a tree, it strikes the front of the neck below the line of the helmet. It exerts a powerful impact across constrained space, which may cause serious crumbling and disintegrating injuries to the cartilage of the larynx and trachea and restrict the airway. Due to the shearing pressures, this might potentially result in laryngotracheal separation. In sports or fights, minor blunt laryngeal injuries can occur. They can cause shearing pressures that may not always appear on inspection, such as hyoid fractures or submucosal endolaryngeal injuries. Due to anatomical defects, airway blockage may develop immediately, or it may take time for patients to experience symptoms resulting from delayed airway obstruction.

Penetrating Laryngeal Injury

Again, the mechanism will determine the severity. Knife wounds, for example, may initially present with minor symptoms of a lower-velocity penetrating injury, but post-injury edema or hematoma may obstruct the airway. High-velocity injuries caused by weapons, such as hunting or military rifle, are generally devastating, fracturing and obliterating laryngeal tissues and supporting systems. In addition to any acute airway difficulties brought on by tissue disturbance or post-injury edema, a relative devascularisation and scarring of these tissues might result in severe long-term constriction.

History and physical

The background information is essential for determining the possibility of laryngeal injury. Patients who have experienced anterior neck trauma should be highly suspected of laryngeal trauma. This covers the mode of injury (blunt or penetrating), the period since the injury, and any other injuries that may have occurred. It is crucial to note the possibility of laryngeal damage in a poly-trauma patient, such as a vehicle accident, because the patient may not be coherent or cooperative. Alarming clinical features of the trauma of the larynx are a history of injury in the anterior part of the neck, husky voice or alteration of voice, pain in the anterior neck, breathlessness, difficulty swallowing, and subcutaneous emphysema.

The actual assessment starts with an estimation of the patient's breathing condition. Follow the emergency trauma assessment: Airway, breathing, circulation. Stridor may be inspiratory, expiratory, or biphasic, and vocal abnormalities may or may not go along with it, depending on the extent of the damage. All patients with neck trauma need to be examined for any accompanying cervical spinal injury. When inspecting the neck, the thyroid cartilage and trachea should be checked for any discomfort, ecchymoses over the anterior neck, subcutaneous emphysema, or loss of the distinctive prominence of the thyroid cartilage, and, if it is possible, checking the voice for hoarseness [14].

Assessment

Setting up a secure airway is the first priority in an emergency case; this may necessitate tracheostomy or orotracheal intubation. Once the patient is stabilized, and the airway and cervical spine are secured, a computed tomography (CT) scan is recommended to check for and assess any laryngeal damage [2,9,15-25].

Flexible Laryngoscopy

The most significant evaluation occurs when a patient's airway appears intact. Tracheostomy and cricothyrotomy should be performed if there is any uncertainty regarding the airway. Flexible laryngoscopy is used to evaluate the interior mucosal tissues of the larynx and upper aerodigestive tracts after the primary and secondary trauma assessments are finished. This can detect edema, which is quite prevalent, and any laryngeal lacerations, hematomas, mucosal tears, or other structural abnormalities could lead to further difficulties. It will demonstrate how serious any mucosal wounds are if any while checking for exposed vocalis muscle or cartilage and assessing the vocal cords' range of motion. For concealed laryngotracheal damage, newly developed vocal fold paresis is concerning, and additional investigation should be started. In the acute environment, it could be challenging to see the subglottis. Although a routine flexible laryngeal exam is encouraging, further testing, such as bronchoscopy in the operating room, may be necessary if the patient's symptoms and manner of damage raise concerns about hidden injury. An asymptomatic patient with a standard flexible laryngeal exam prior to imaging or further workup may not require intubation or tracheostomy.

Computed Tomography (CT)

Imaging should only be employed when it is safe and influences the management strategy. Imaging is probably not beneficial for patients with a positive history, clinical picture, routine review, and laryngoscopy. Similarly, people with an observable injury won't probably benefit because surgery will be necessary. Patients with a steady airway and a positive assessment with a strong clinical suspicion for undiscovered harm may benefit from computed tomography (CT) scan. The cartilaginous and bony components of the hyoid and larynx can be seen on a non-contrasted CT of the neck, as well as any minor or nondisplaced fractures that may need stabilization.

Esophagram

Four to six percent of laryngeal fractures will also result in oesophageal damage. Although esophagrams are rarely used in acute situations, they can be helpful if suspected of chronic damage. The best approach is a proper evaluation under anesthesia after securing the airway if there is a doubt of oesophageal damage concerning laryngeal injury. Procedures include direct laryngoscopy, esophagoscopy, and maybe an open neck evaluation in the operating room.

Chest X-ray

In a stable condition of the patient with a secured airway, a portable roentgenogram may typically be used to collect this information in the case of a poly-trauma patient with doubtful neck trauma. On a clinical examination, subcutaneous emphysema may signal a laryngotracheal injury and chest wall damage.

Treatment/Management

The initial step in treating laryngeal injuries is recognizing and establishing an airway. "Does the patient have a stable airway?" is the initial decision-making criterion. If the patient is talking normally, the airway is at least patent; but may not be steady [26].

There have been reports of hoarseness, dysphagia, odynophagia, anterior neck discomfort, dyspnea, stridor, coughing, and hemoptysis as laryngeal fracture symptoms. Clinical signs include cracking on palpation, ecchymosis, hematoma, neck wound, pain, and surgical emphysema [2,7,17,19,27-33].

Breathlessness, neck hematoma, severe hemorrhage, subcutaneous neck emphysema, stridor, soreness of throat, expectoration of blood, thrill or bruit, and altered throat architecture all enhance the need for intubation, cricothyroidotomy, or tracheotomy. Tracheostomy or cricothyrotomy should be done right away for anyone with an evident larynx fracture, stridor with elevated breathing labor, or an imminent airway blockage.

In 2014, Schaefer examined 90 years' worth of literature on acute trauma of the larynx [34]. Based on this literature evaluation and his clinical knowledge, he suggested the management strategy described below:

Impending airway obstruction: Competent airway therapy leads to a cricothyrotomy, tracheostomy, or intubation. After that, direct laryngoscopy and oesophageal scoping are performed on every patient. Following laryngoscopy and esophagoscopy, the following should be done (Table 2).

**Table 2:** Treatment modality/management for the type of injury ORIF: Open reduction and internal fixation

Type of Injury	Treatment modality/ Management
Normal endolarynx or fracture, less trauma of mucosa.	Monitoring
Fracture of the thyroid or cricoid with an uninjured endolarynx.	Examination of throat, open reduction, and internal fixation (ORIF) of skeletal fractures of the larynx with plating without thyrotomy.
Unsteady fractures or anterior commissure interruption, or major mucosal lacerations.	ORIF of fractures, correction of laceration of mucosa, and stent of endolarynx or keeper of the lumen.
Steady fractures of the larynx, uninterrupted anterior commissure, change of mucosa which is less severe.	Examination of the throat, ORIF of skeletal fractures of the larynx with plating thyrotomy, primary closure of lacerations.

Stable airway: Laryngoscopy with flexible fiber optics and neck computed tomography. Depending on availability and local expertise, laryngeal electromyography and videostroboscopy may also be employed. The conclusions of these investigations guide treatment (Table 3).

**Table 3:** Treatment for the type of injury

Type of Injury	Treatment Modality/Management
Normal endolarynx in which reversible trauma to mucosa may or may not be present without fracture	Monitoring.
Interruption of cartilage or endolarynx	Tracheostomy or intubation, direct laryngoscopy and esophagoscopy, throat examination, and correction of findings like "impending airway obstruction”.

The best outcomes are obtained when airway injuries are treated within 24 hours.

Medical management/close observation:* *Close monitoring could be sufficient in cases with Schaefer type 1 and 2 injuries. To treat edema, dexamethasone is administered intravenously in addition to inhalational (nebulized) steroids. For the first 24 hours at least, serial flexible laryngoscopy and airway monitoring with pulse oximetry should be performed. This could be all that's necessary in cases like these when there is a normal or almost normal mucosal layer and a slight chance of unfavorable laryngeal scarring.

Surgical treatment: Surgery is necessary for all other situations, and thorough anesthetic examinations of the esophagus and larynx with direct laryngoscopy and endoscopy, respectively. To cover all muscle and cartilage and prevent scarring, endoscopic repair may be tried if mucosal lacerations are relatively mild to moderate. Significant mucosal lacerations, unstable fractures, or displaced fractures will necessitate an open neck examination and probably a thyrotomy for treatment. The mucosa should be mended first to provide a covering and prevent scarring or webbing. Any oesophageal injuries should be addressed, and finally, any laryngeal fractures should be reduced and corrected. Repairing soft tissue and skin should come last. A formal thyrotomy may not be necessary if the intralaryngeal lesions are mild, but this method should be customized to the specific injuries that are present. A stent could be essential if there are extensive intralaryngeal mucosal lesions; however, such devices should only be used rationally because they can also result in scarring.

These patients should at least spend the first night in the critical care unit after surgery under strict observation. Many patients will need tracheostomy until their larynx has fully recovered and can undergo a formal evaluation with the help of a speech therapist. Due to the significant danger of aspiration, nasogastric or gastrostomy tubes should be used to feed the patients until the larynx has healed.

Prognosis

Laryngotracheal injuries may be fatal; thus, it is essential to manage the airway immediately. Missing a severe laryngeal injury can cause airway blockage, which can result in death. Patients with laryngeal or tracheal abrasions or minor tears are treated conservatively with constant monitoring, frequent scopes, and steroids. Early airway treatment benefits laryngeal fracture patients since it increases healing times. Early identification and therapy improve the voice and airway results of patients.

Complications

The severity of complications might range from highly minor to catastrophic. As the intralaryngeal mucosa heals, any lesion to it is likely to have some granulation tissue. This can occasionally be rather serious and result in scarring or, extremely rarely, an obstructive tumor. Cicatrix development is a terrifying complication, and the best prevention is quick mucosal healing and avoiding laryngeal muscle or exposed cartilage. Long-term tracheostomy reliance may occur if scarring is severe or persistent. Wound healing complications such as recurrent tracheo- or laryngo-cutaneous fistulae are fortunately uncommon, although they can occur. Unrecognized oesophageal damage is a potentially fatal side effect. Vocal fold paresis or paralysis evident at the presentation following trauma would commonly improve, but it may take up to a year. Moreover, such an injury may be persistent and need additional care. The superior or recurrent laryngeal nerves might also sustain damage during recovery, leading to dysphonia or aspiration and necessitating additional medical care.

Postoperative and rehabilitation care

All traumatic laryngeal injuries require hospitalization for observation. Minor mucosal injuries, nondisplaced fractures, and hematomas are a few examples of laryngotracheal injuries that can be treated conservatively with head elevation, humidified air, analgesics, steroids, vocal rest, and a clean diet. The long-term therapy objective for these individuals is restoring their voice and swallowing system. In addition to the possibility of longer-term tracheostomy or gastrostomy reliance, more serious injuries may necessitate lengthy speech and swallowing treatment [1].

Consultations

To help diagnose and treat laryngeal trauma, head and neck surgeons should be consulted in cases of suspected laryngeal injuries. If such knowledge is not accessible, a surgeon and anesthesiologist skilled in managing complicated airways are crucial. The doctor with the most expertise doing such treatments should conduct an elective surgical airway in an emergency circumstance if there is any doubt about the stability of the airway (often through an awake tracheostomy).

Deterrence and patient education

Patients who have suffered a neck injury, whether from a car accident, a gunshot, an attempted hanging, a neck knife, or an attack, should go to the emergency room for examination because laryngeal or tracheal injuries are significant injuries that can be fatal. Serious delayed complications might happen even if the patient is currently asymptomatic. A tracheostomy is typically necessary for people who have respiratory difficulties. Intubation attempts must be postponed until a more comprehensive image of the patient's upper airway is obtained; if this is not available, a tracheostomy must be done instead. Patients who are stable and have had no severe trauma can require imaging, monitoring, or hospitalization.

Pearls and other issues

The trauma of the larynx and trachea must be managed to secure the airway since they have a high fatality rate. A delayed airway compromise is possible. In the trauma of the larynx and trachea, the recommended airway is the tracheostomy. A CT scan or other further imaging may be required for stable injuries. Unstable wounds require surgical treatment. Do not wait to contact a surgeon for the trauma of the larynx and trachea [2].

Case series

Case 1

A 24-year-old female came to the emergency department with a history of attack by a knife on her neck five hours ago. The father was the informant. There were several lacerations on the neck, face, and shoulder, with the greatest incision reaching around 15x3 cm and stretching from the right sternocleidomastoid to the left sternocleidomastoid horizontally and obliquely towards the left angle of the mandible. The trachea was visible, and the defect allowed air to escape through. There was no visible cervical spine damage, no free bleeding, and no neck swelling. The patient was immediately sent to the operating room, and the airway was secured by performing a low tracheostomy below the defect. After exploring the neck wound, the neck's primary vasculature was discovered to be in good condition. After giving the wound a thorough saline wash, debridement and primary closure were performed. The neck wound was first closed in layers, with the skin being sutured with 3-0 Ethilon, and a defect above the anterior trachea was identified. The patient had a nasogastric tube placed, and antibiotic therapy was started. The patient was able to speak after having the sutures removed and the tracheostomy tube replaced with Fuller's tracheostomy tube no. 30 on day 14. On the 21^st^ day, the nasogastric tube was withdrawn. After that, the patient was released, and a week later, a follow-up was conducted [35].

Case 2

A 34-year-old female came to the emergency department with an unknown type of injury across the neck. Her husband was the informant. The laceration had uneven edges and ranged from the right sternocleidomastoid to the left sternocleidomastoid. Tissue was lost, exposing the trachea and larynx. The tracheal defect was causing an air leak. The patient had a peripheral capillary oxygen saturation (SpO2) of 96% and was hemodynamically stable. The patient was then sent to the operation theatre. After performing a tracheostomy, it was discovered on examination of the wound above that the anterior cricoid cartilage lamina and the first tracheal ring had been lost. There was damage to the vocal cords, a cut to the strap muscles, the main vessels of the neck were intact, and there was no neck swelling. A thorough saline wash was performed, followed by debridement of the wound and primary closure. The tracheal and cricoid cartilage defects were fixed using the patient's auricular conchal cartilage and 3-0 vicryl sutures. The remaining injury was stitched up in layers. After then, the patient was shifted to a ventilator. Forty-eight hours after the surgery, the patient passed away.

Case 3

A three-year-old boy with cut and lacerated wounds on the anterior area of his neck from an unknown animal attack was brought to the hospital by his parents. Parents served as the informant. At the time of the examination, the patient was sleepy. On 4 liters of oxygen, SpO2 was 94%. The child was sent to the operation theatre, where he was intubated using 4.5 sized uncuffed endotracheal tube. Major vessels were unharmed, strap muscles and thyroid membranes were torn, and thyroid cartilage was lacerated on the left lateral aspect. The whole body had several minor abrasions. After a thorough saline wash, the wound was debrided, and the main closure procedure was carried out. After treating the cricothyroid laceration, the neck wound was stitched up in layers, the strap muscles were closed with 3-0 Vicryl, the neck wound was stitched up with 3-0 Monocryl, and the patient was placed on a ventilator for 48 hours. The patient died 48 hours after the operation.

Case 4

A 24-year-old male came to the emergency department with a history of attack by a knife on his neck two hours ago. The informant was his brother. On inspection, a laceration spanning from the anterior border of one sternocleidomastoid muscle to the other, measuring approximately 7x5 cm, platysma and strap muscles were split in the midline, and thyroid and cricoid cartilage were exposed. Vocal cords were visible due to a thyroid cartilage defect in the center. After the injury, he vomited thrice. He had a steady hemodynamic state. He was quickly transferred to the operating room, followed by awake intubation through exposed vocal cords under local anesthesia; a tracheostomy was done to secure the airway, followed by a comprehensive examination of the lesion. The neck's major vessels were found intact, and no swelling was present. The thyroid cartilage was first closed utilizing 3-0 Vicryl, and the laceration was stitched up in layers. To close the skin, 3-0 Ethilon was used. After being placed on a ventilator for 48 hours, the patient was eventually allowed to go home.

Case 5

A 45-year-old female came to the emergency department with a history of self-trauma by a blade to her neck one hour ago. The informant was her daughter. The patient was a known leprosy case. On inspection, two distinct lacerations were found, the bottom one deep with exposed thyroid cartilage and a defect and the upper one superficial with an air leak. The lower laceration was 6x3 cm in size and extended from the anterior edge of the left sternocleidomastoid muscle across the midline. Her hemodynamics were steady. Two previous incidents of vomiting following trauma were documented. Ryle's tube was inserted. She was sent to the operating room. The wound was then carefully examined. There was no swelling in the neck, and the neck's prominent veins were sound. Utilizing 3-0 Vicryl, the thyroid cartilage was first closed, and the incision was stitched up in layers. 3-0 Ethilon was used to close the skin. The patient was allowed to go home [35].

Case 6

A 28-year-old male presented to the emergency unit with complaints of neck injury because of an accident. He was riding his bike when the vehicle in front of him suddenly applied brakes. Because of this, the anterior part of the neck struck against a horizontal metallic rod of a ladder projecting from the back of the vehicle. As a result, the anterior part of the neck got injured. He had trouble breathing and could not talk when he was taken to the emergency room. He was found to have surgical emphysema throughout his chest and neck and a lacerated incision over his left parotid. No edema, laceration, or bruising around the larynx was found to indicate any external trauma. In a lying-down posture, the patient suffered stridor and could not talk. A tracheostomy was performed since the damage raised the possibility of a laryngeal injury. Pneumomediastinum was seen on the chest X-ray. The salivary collection that had formed over the parotid wound was drained, and a pressure dressing was placed. A CT neck was done following the patient's overall stable condition. It showed a comminuted fracture of the left lamina of the thyroid cartilage with two fracture lines, one vertical in the midline displacing the left lamina and another horizontal in the left thyroid lamina, along with a chip fracture of cricoid displacing the arytenoid anteromedially. Even on deep inspiration, the glottic chink seemed narrower during preoperative video laryngoscopy, and the space between the anterior commissure and vocal cords had shrunk. The laryngeal damage was rated according to Schaefer's classification for severity of the laryngeal injury, and it was Grade IV based on endoscopic and CT findings.

The patient was brought to the operating theatre, where the thyroid cartilage underwent an open reduction and internal fixation (ORIF) with 4-0 prolene sutures. Due to the fragile cartilage and tight screw fixation being impossible, plates could not be fixed. Vicryl 6-0 was used to stitch mucosal tears. Vocal cords had retracted and were now sutured with 4-0 Vicryl to the external perichondrium of the thyroid cartilage. Endolaryngeal stenting was carried out using a 3.5 mm long, 6 mm external diameter polyvinyl chloride (PVC) endotracheal tube, with the top end closed with sutures to avoid aspiration. A clamp was used to reduce the diameter of the tube's section that corresponded to the true and false vocal cords. The stent was positioned to pass superiorly through the true and false vocal cords and inferiorly to the first tracheal ring and secured with 4-0 prolene sutures that traversed the stent superiorly through the laryngeal ventricle and inferiorly through the cricothyroid membrane. The sutures were crossed through the strap muscles laterally and then through a button connected to the skin. It will be removed when the stent is taken out. To reduce secretions and edema, injectable antibiotics such as piperacillin and tazobactam, gentamicin, and clindamycin were administered together with dexamethasone and nebulized acetylcysteine and adrenalin. The stent was removed by direct laryngoscopy after 11 days. Two weeks after the incident, a videostroboscopy revealed limited left vocal cord movement, a normal anterior commissure, and a moderately patent glottic chink. Initial swallowing techniques helped him overcome minor aspiration difficulties. After six months, despite continuing slight hoarseness of voice for which he is undergoing speech therapy, there are no discernible swallowing or breathing issues [36].

## Conclusions

Even though laryngotracheal wounds are infrequently observed, they have a significant mortality rate. These wounds can be blunt or piercing and can damage the supraglottic, glottic, or infraglottic regions. When a patient with a laryngeal injury arrives in the emergency room, the airway should be secured first. According to a review of the mentioned cases, primary closure is advised if the precise nature of the damage is known and the prognosis is good. These cases demonstrate the need to collect a thorough history and maintain a high degree of suspicion for laryngeal fracture in a blunt trauma patient with pneumomediastinum and subcutaneous emphysema. A CT scan should rule out upper airway injuries such as a laryngeal fracture. Laryngotracheal damage to patients' airways might be challenging to control. Depending on local knowledge and accessibility, emergency department doctors, anesthesiologists, trauma surgeons, otolaryngologists, and respiratory therapists may help with airway management. The prognosis for long-term voice and airway outcomes is improved by early recognition of the injury and timely intervention.
